# Evaluating the Phagocytic Index of Peripheral Leukocytes in Endometriosis by Plasma Experiments

**DOI:** 10.3390/medicina58070925

**Published:** 2022-07-12

**Authors:** Luca Lukács, Anna Rebeka Kovács, László Pál, Sándor Szűcs, Rudolf Lampé

**Affiliations:** 1 Department of Obstetrics and Gynecology, Faculty of Medicine, University of Debrecen, 4031 Debrecen, Hungary; lukacs.luca@med.unideb.hu (L.L.); kovacsanna931@gmail.com (A.R.K.); 2 Department of Public Health and Epidemiology, Faculty of Medicine, University of Debrecen, 4028 Debrecen, Hungary; pal.laszlo@med.unideb.hu (L.P.); szucs.sandor@med.unideb.hu (S.S.)

**Keywords:** endometriosis, innate immunity, phagocyte function, plasma experiment, monocyte, neutrophil granulocyte

## Abstract

*Background and Objectives*: Endometriosis is a benign, chronic disease, that negatively influences the quality of life of affected women and is responsible for a remarkable amount of infertility. The pathophysiology of the disease is still not clarified, but the insufficient immune surveillance plays a significant role in it. The phagocyte function of innate immune cells may play a role in the elimination of ectopic endometrium. The purpose of this study is to examine the phagocyte function of neutrophil granulocytes and monocytes, incubated in heat-inactivated and not-inactivated plasma samples from healthy women and from women with endometriosis before and after the surgical treatment. *Materials and Methods*: Blood samples were collected from eight preoperative and eight postoperative patients with endometriosis before and after the surgical treatment, and from 16 healthy patients as controls. Neutrophil granulocytes, monocytes and blood plasma samples were isolated. Cells were incubated in different plasma samples, and the phagocytic index was determined with a fluorescence microscope. *Results:* The phagocytic index of granulocytes and monocytes isolated from patients with endometriosis was significantly decreased compared to healthy women after the cells were incubated in their own plasma. Preoperatively isolated cells from patients with endometriosis demonstrated an improved phagocyte function after incubating them in plasma samples from healthy controls. In contrast, the phagocytic activity of cells from healthy women significantly reduced after being incubated in the plasma of preoperative endometriosis patients. The heat-inactivation of plasma samples did not affect the results. *Conclusions:* Active endometriosis lesions may produce heat-stable systemic immunomodulatory factors, which reduced the phagocyte function of peripheral monocytes and neutrophil granulocytes. The phagocyte function of these cells can be normalized after the complete surgical removal of endometriosis, which then demonstrates similar values as in healthy women.

## 1. Introduction

Endometriosis is a chronic, systemic, estrogen-dependent inflammatory disease in which endometrial glands and stromal tissues emerge outside the uterine cavity and function synchronously with the menstrual cycle at their location. The complaints caused by endometriosis are dysmenorrhea, dyspareunia, infertility, and chronic pelvic pain. Other complaints such as constipation, diarrhea, and dysuria may occur depending on the location of the endometriosis [1,2]. The disease affects at least 10% of women of reproductive age and the related pain and infertility significantly impair their quality of life. Despite decades of research, the pathophysiology of endometriosis is not fully clarified, and the assumptions contain several contradictions [3,4,5].

During pregnancy, endometriosis is considered to be absent, as a consequence of high progesterone levels, but recently more and more evidence has been revealed about the adverse obstetrical outcomes of the disease. Women with endometriosis have higher risks for preterm delivery and neonatal admission to neonatal intensive care unit; moreover, if endometriosis is associated with severe adenomyosis, as is known as a frequent situation, the risk of placenta previa and cesarean section is increased significantly. The possible causes of these complications are chronic inflammation, adhesions, and progesterone resistance related to endometriosis [6,7].

Several efforts were made to look for non-invasive methods for the replacement of histological verification in recent years. Identification of the metabolomics profile of patients could be one solution in the future, as studies demonstrate significantly higher levels of β-hydroxybutyric acid and glutamine, a decreased amount of tryptophan and altered nitrogen, pyrimidine, glutamine, and glutamate metabolism [8]. Although numerous blood markers have been studied, surgery is still the gold standard of diagnosis and treatment. Mild elevation of the CA-125 tumor marker level and changes in the mean platelet volume and neutrophil-to-lymphocyte ratio has been observed in endometriosis, but the results are conflicting [9,10].

For a better description of the pathophysiology, several theories have been established so far, but none of them explains it in its entirety. The theory of retrograde menstruation has gained support from observations from surgical explorations during menstruation when in 90% of the cases menstrual blood can be observed in the small pelvis. However, this phenomenon occurs in most women without endometriosis as well. Vascular and lymphatic dissemination, mesothelium, stem cells, Mullerian rest or genetic components may play a role in the presence of extrauterine endometrial tissue [11,12]. Transition of intestinal microbes, caused by altered intestinal absorption, may also contribute to the development of chronic pelvic pain as a consequence of a disturbed global immune response [13,14]. Nowadays, most studies focus on the immunological background of endometriosis, and it can be concluded that the modified immunological function may contribute to the endometrial cells for adhering and growing outside of the uterine cavity, initiating neovascularization, and provoking a significant inflammatory response in their surroundings [15].

Representatives of the innate immune system, the neutrophil granulocytes and monocytes capable of phagocytosis, are within a process in which these cells can ingest and eliminate particles such as microorganisms, foreign substances, and apoptotic cells. Phagocytosis seems to be a vital function not only for these reasons but also due to its contribution in tissue homeostasis and even in pregnancy [16,17]. Despite the importance of phagocytosis, its role is rarely studied in endometriosis. In our previous work, we described that phagocyte function of neutrophil granulocytes and monocytes is decreased in endometriosis compared to healthy women and after surgical removal of the lesion the phagocyte function is normalized [18].

The main aim of this work is to provide a more detailed understanding of the role of phagocytes in the pathogenesis of endometriosis and to examine the effect of different plasma samples on phagocyte function.

## 2. Materials and Methods

### 2.1. Study Population

After written informed consent and with the permission of the Ethics Committee, peripheral blood samples were collected in vacutainer test tubes containing EDTA from 8 women with endometriosis before and from the same patients after the surgery, and from 16 healthy women as a control. The patients were included in this experimental research study between 2020 and 2021. Inclusion criteria was the regular menstrual cycle and normal blood count. Any chronic systemic or suspected immunological disease, smoking, immunomodulatory or hormonal therapy served as exclusion criteria. Peripheral venous blood samples were collected in the morning before surgery and seven days after the surgical intervention from the same patients. Endometriosis was evaluated and completely removed in all cases during laparoscopic surgery and confirmed histologically. All women had stage I–II disease according to the Revised American Society for Reproductive Medicine classification of endometriosis [19].

### 2.2. Preparation of Opsonized and FITC-Labeled Zymosan Particles

For the proper evaluation of phagocytosis, fluorescein isothiocyanate (FITC)-labeled Zymosan particles were used, as described previously [20]. With carbonate buffer incubation, the particles (1 × 10^8^ /mL), at pH 9.6, containing FITC at a final concentration of 0.01 mg/mL for 60 min at 37 °C, were produced. Then, they were washed and opsonized in Hanks’ solution containing 50% human AB serum at 37 °C for 30 min. The labeled and opsonized particles (FITC-OZ) were stored at −20 °C in Hanks’ solution (3 × 10^7^/mL) until the incubation with cell culture chambers, where the phagocytosis reaction happened.

### 2.3. Separation of Plasma Fractions, Granulocytes, and Monocytes

In all groups, plasma fractionation occurred with centrifugation of the blood samples at 800× *g* at 20 °C for 10 min. After this procedure, we divided all plasma samples into two subgroups, and one of the subgroups was inactivated in heated water (56 °C, 30 min) to exclude the possible effect of complement activity on phagocyte function and for the observation of whether the examined plasma factors are heat resistant or heat labile, while the other subgroup was incubated at room-temperature at this time. The separation of monocytes and neutrophil granulocytes occurred with different Ficoll gradients. Blood samples were mixed with an equal volume of Hanks’ solution (pH 7.4), layered on the top of a discontinuous Ficoll gradient (1.077 and 1.119 g/mL) and centrifuged at 400× *g* at 20 °C for 30 min. Monocytes and neutrophil granulocytes were collected from the top and interface of the Ficoll layers, respectively, then washed with Hanks’ solution. The viability of the cells was determined by a trypan blue exclusion test and found to be 96–98%. The purity of granulocyte and monocyte suspensions varied between 95% and 98%, as judged by their morphology under a microscope. Lysis of the red blood cells was not necessary because of the lack of contamination [21].

### 2.4. Plasma Incubation

All plasma experiments were performed with both heat-inactivated and non-inactivated plasma samples. To observe the effect of foreign plasma on the phagocyte function, the cells of each group were treated in their own (autologous) plasma and in the plasma of another individual (heterologous) from the same group.

Preoperatively harvested monocytes and neutrophil granulocytes from women with endometriosis were incubated in plasma from healthy women and in plasma from preoperative women with endometriosis (autologous and heterologous). 

In an analogous fashion, postoperatively separated monocytes and neutrophil granulocytes from women with endometriosis were incubated in plasma from healthy women and in plasma from postoperative women with endometriosis (autologous and heterologous). 

Cells from healthy women were incubated in autologous and heterologous plasma, and also in pre- and postoperatively isolated plasma from women with endometriosis. 

After incubation in plasma samples (37 °C for 60 min), the cells were washed twice using Hank’s solution and the samples were centrifuged.

### 2.5. Cell Number Adjustment and Adhesion

Adhesion was performed in a cell culture chamber using Hank’s solution, which contained inactivated AB-human serum, and prevented the activation of cells on the glass surface. Each chamber was eventually loaded with 250 µL of cell suspension (previously incubated in the appropriate blood plasma) and 50 µL of Hank’s solution containing inactivated AB-human serum and fixed for 30 min at room temperature. This was followed by additional washings with Hank’s solution to rinse away the non-adhered cells.

### 2.6. Phagocytic Activity, Cell Membrane, and Nucleus of Cells

**Figure 1:**
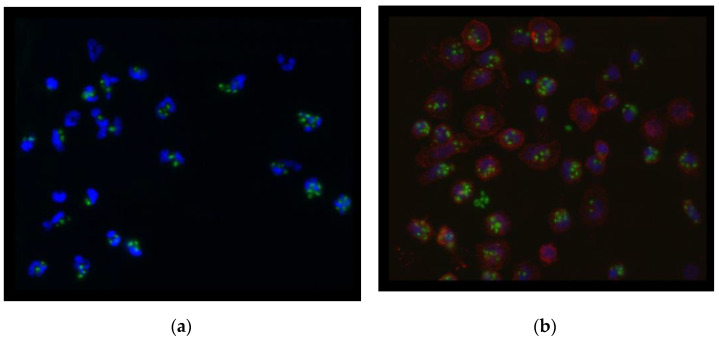
Fluorescence microscopic view (blue: nucleus of cells, green: Zymosan particles, red: cell membrane of monocytes) of neutrophil granulocytes (**a**) and monocytes (**b**) with phagocyted zymosan particles.

A 300 µL suspension of previously described FITC-labeled and opsonized zymosan particles was added after thawing and diluting each chamber of adhered cells (60 min, 37 °C air with 5% CO_2_ and 100% relative humidity). The fluorescence of non-engulfed particles was neutralized using trypan blue dye. After chamber separation, plates containing both monocytes and granulocytes were fixed in 4% paraformaldehyde solution for 30 min. Monocytes were labeled with CD14+ monocyte-specific antibodies. This was followed by rinsing before labeling with fluorescently labeled (DyLight 594) secondary antibodies against mouse IgG. The nuclei of both neutrophil granulocytes and monocytes were labeled with a mounting medium containing 4′,6-diamidino-2-phenylindole. The amount of engulfed FITZ-OZ per cell was determined in randomly selected fields of view using an Axioplan fluorescence microscope (Zeiss, Oberkochen, Germany). The neutrophil granulocytes were identified through their morphological characteristics and the monocytes with red fluorescent cell membranes provided a clue for cell identification [22]. The final view of the cells is demonstrated on Figure 1a,b. 

The phagocytic index, the average number of ingested particles/cells, was calculated and presented as mean ± standard deviation.

### 2.7. Statistical Analysis

The Shapiro–Wilk test was used to verify normal distribution of the data. The one-way analysis of variance (ANOVA) was used to analyze clinical data from the study participants. The repeated measures ANOVA was used to compare the phagocytic index obtained after treatment of monocytes and granulocytes within the same subjects (patients with endometriosis or healthy subjects) with different plasma varieties (autologous, heterologous, pre- and postoperative). To compare the results after the treatment with inactivated and non-inactivated plasma, the Student’s two-sample *t*-test was used for each plasma combination. Values of *p* < 0.05 were considered to indicate statistical significance.

## 3. Results

### 3.1. Clinical Data of Participants

Table 1 describes the clinical data of patients with endometriosis and healthy women. No statistical difference was found between the groups in age, body mass index, gravidity, or parity at the time of blood sampling.

### 3.2. Phagocytic Index of Neutrophil Granulocytes and Monocytes from Healthy Women

**Figure 2:**
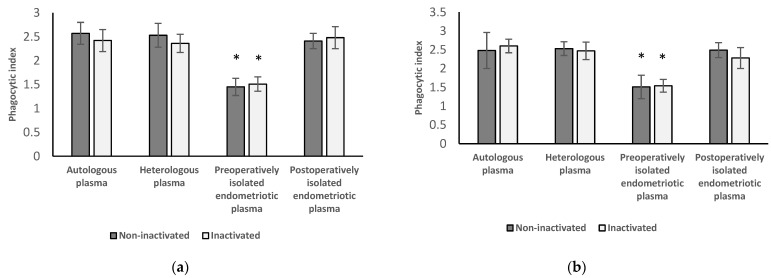
Phagocytic index of neutrophil granulocytes (**a**) and monocytes (**b**) of healthy women followed by the treatment with autologous and heterologous plasma from healthy women and with plasma separated preoperatively and postoperatively from patients with endometriosis. Experiments were performed with inactivated and non-inactivated plasma samples in each group. * *p* < 0.001 compared with autologous, heterologous, and postoperative group.

Figure 2 shows the results of neutrophil granulocytes (a) and monocytes (b) from healthy women followed by the treatment with autologous and heterologous plasma from healthy women and with plasma separated preoperatively and postoperatively from patients with endometriosis.

#### 3.2.1. Results of Neutrophil Granulocytes from Healthy Women

After the incubation of these neutrophil granulocytes, separated from healthy women, in preoperatively obtained inactivated (1.51 ± 0.18) or non-inactivated (1.45 ± 0.15) plasma from women with endometriosis, the phagocytic index of the cells decreased significantly (*p* < 0.001) compared to those treated with inactivated (2.42 ± 0.23) and non-inactivated (2.57 ± 0.26) autologous and heterologous plasma (inactivated: 2.36 ± 0.25; non-inactivated: 2.53 ± 0.19) from healthy women. The phagocytic index of the examined cells did not differ significantly (inactivated: *p* = 0.368 and 0.238; non-inactivated: *p* = 0.053 and 0.344) after incubated in inactivated and non-inactivated autologous or heterologous plasma from healthy women compared to the results following the incubation of these cells in plasma from women with endometriosis collected postoperatively (inactivated: 2.48 ± 0.16; non-inactivated: 2.41 ± 0.23).

#### 3.2.2. Results of Monocytes Isolated from Healthy Women

Phagocytic index of these monocytes after the treatment with plasma from patients with endometriosis before the surgery (inactivated: 1.54 ± 0.17; non-inactivated: 1.51 ± 0.31) are significantly decreased (inactivated: *p* < 0.001; non-inactivated: *p* < 0.001) compared to the phagocytic index of the cells that are treated with autologous (inactivated: 2.60 ± 0.18; non-inactivated: 2.48 ± 0.48) or heterologous plasma (inactivated: 2.47 ± 0.23; non-inactivated: 2.53 ± 0.18) from healthy women. Phagocytic index of the examined cells did not differ significantly (inactivated: *p* = 0.05 and 0.482; non-inactivated: *p* = 0.929 and 0.581) after incubating in inactivated and non-inactivated autologous or heterologous plasma from healthy women compared to the results following the incubation of these cells in plasma from women with endometriosis postoperatively (inactivated: 2.28 ± 0.28; non-inactivated: 2.49 ± 0.20). 

#### 3.2.3. Results from Inactivated versus Non-Inactivated and Heterologous versus Autologous Plasma of Healthy Women

No significant difference can be found between the results after the treatment with inactivated and non-inactivated plasma in either group. There was also no significant difference in phagocytic function if the cells were incubated in heterologous plasma versus autologous plasma.

### 3.3. Phagocytic Index of Neutrophil Granulocytes and Monocytes from Women with Endometriosis before Surgery

**Figure 3:**
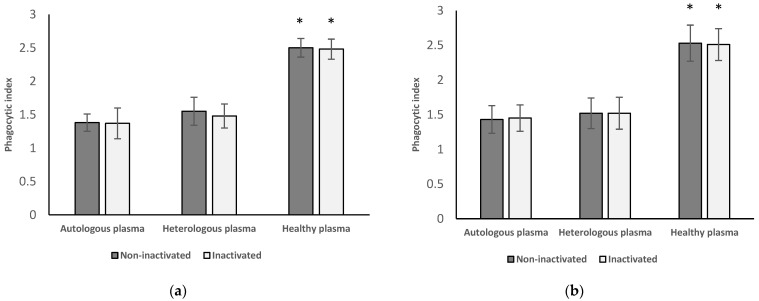
Phagocytic index of neutrophil granulocytes (**a**) and monocytes (**b**) of women with endometriosis before surgery followed by the treatment with autologous and heterologous plasma from the same group of patients and with plasma from women with endometriosis after surgery. Experiments were performed with inactivated and non-inactivated plasma samples in each group. * *p* < 0.001 compared with autologous and heterologous group.

Figure 3 shows the results of neutrophil granulocytes (a) and monocytes (b) from women with endometriosis before surgery followed by the treatment with autologous and heterologous plasma from the same patient group and plasma from healthy women.

#### 3.3.1. Results of Neutrophil Granulocytes Preoperatively Separated from Women with Endometriosis

After incubating these neutrophil granulocytes with inactivated (1.37 ± 0.23) and non-inactivated autologous (1.38 ± 0.13) and heterologous plasma (inactivated: 1.48 ± 0.18; non-inactivated: 1.55 ± 0.21) plasma from women with endometriosis before surgery, phagocytic index of the cells decreased significantly (*p* < 0.001) compared to those that were treated with inactivated (2.48 ± 0.15) and non-inactivated (2.5 ± 0.14) plasma from healthy women.

#### 3.3.2. Results of Monocytes Preoperatively Isolated from Women with Endometriosis

Phagocytic index of these monocytes after the treatment with inactivated (1.45 ± 0.19) and non-inactivated (1.43 ± 0.20) autologous and heterologous (inactivated: 1.52 ± 0.23; non-inactivated: 1.52 ± 0.22) plasma from women with endometriosis before surgery is decreased significantly (*p* < 0.001) compared to those that were treated with inactivated and non-inactivated plasma from healthy women (inactivated: 2.51 ± 0.23; non-inactivated: 2.53 ± 0.26). 

#### 3.3.3. Results from Inactivated versus Non-Inactivated and Heterologous versus Autologous Plasma from Preoperative Women

No significant difference can be found between the results after the treatment with inactivated and non-inactivated plasma in either group. There was also no significant difference in phagocytic function if the cells were incubated in heterologous plasma versus autologous plasma.

### 3.4. Phagocytic Index of Neutrophil Granulocytes and Monocytes from Women with Endometriosis after Surgery

**Figure 4:**
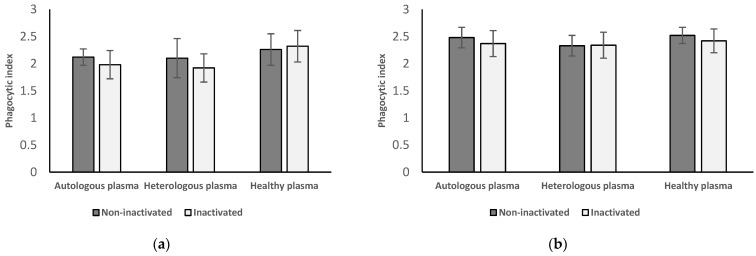
Phagocytic index of neutrophil granulocytes (**a**) and monocytes (**b**) of women with endometriosis after surgery followed by the treatment with autologous and heterologous plasma from the same group of patients and with plasma from healthy women. Experiments were performed with inactivated and non-inactivated plasma samples in each group. No significant difference was found between the groups.

Figure 4 shows the results of neutrophil granulocytes (a) and monocytes (b) from women with endometriosis after surgery followed by the treatment with autologous and heterologous plasma from the same patient group and plasma separated from healthy women.

#### 3.4.1. Results of Neutrophil Granulocytes Postoperatively Separated from Women with Endometriosis

After incubating these neutrophil granulocytes with inactivated (1.98 ± 0.26) and non-inactivated autologous (2.12 ± 0.15) and heterologous plasma (inactivated: 1.92 ± 0.26; non-inactivated: 2.1 ± 0.36) plasma from women with endometriosis after surgery, phagocytic index of the cells shows no significant difference (inactivated: *p* = 0.655 and 0.050; non-inactivated: *p* = 0.504 and 0.845) compared to those that were treated with inactivated (2.32 ± 0.29) and non-inactivated (2.26 ± 0.29) plasma from healthy women. 

#### 3.4.2. Results of Monocytes Postoperatively Isolated from Women with Endometriosis

The phagocytic index of these monocytes after the treatment with inactivated (2.37 ± 0.24) and non-inactivated (2.48 ± 0.19) autologous and heterologous (inactivated: 2.34 ± 0.24; non-inactivated: 2.33 ± 0.19) plasma from women with endometriosis after surgery does not change significantly (inactivated: *p* = 0.517 and 0.300; non-inactivated: *p* = 0.869 and 0.072) compared to those that were treated with inactivated and non-inactivated plasma from healthy women (inactivated: 2.42 ± 0.22; non-inactivated: 2.52 ± 0.15). 

#### 3.4.3. Results from Inactivated vs. Non-Inactivated Plasma and Heterologous vs. Autologous Plasma Regarding Postoperative Results

No significant difference can be found between the results after the treatment with inactivated and non-inactivated plasma in either group. There was also no significant difference in phagocytic function if the cells were incubated in heterologous plasma versus autologous plasma.

## 4. Discussion

With its prevalence currently at around 10% and on the rise among women of childbearing potential, endometriosis is a major public health problem worldwide; moreover, the disease may be responsible for up to 50% of patients with infertility and/or abdominal pain [23]. In our current knowledge, multiple factors are suspected to play a role in the etiopathogenesis of the disease. Until a better understanding is reached, endometriosis remains an underdiagnosed disorder with significant associated morbidity [24].

Alterations of the innate and acquired immunity as well as attenuated immunosurveillance seem to play a paramount role in the pathogenesis of endometriosis in the abdominal cavity and systemically through blood vessels, where endometrial tissue can adhere, grow, and multiply itself outside the uterine cavity and can cause inflammatory changes through the production of different immunomodulatory factors and disturbed function of immune cells. Despite intense research, the process that is supposed to eliminate the ectopic endometrial tissue form healthy women is still not known; however, there are some data regarding acquired and innate immune system both systemically and the peritoneal local environment in the affected areas by endometriosis.

The available data about the function of adaptive immune system shows encouraging signs in the recent years. Due to the results, both in the peritoneal environment and in the peripheral blood, there is a significant increase in the number of NK cells and T lymphocytes; moreover, their cytotoxicity function is reduced, which is thought to be related to substances found in the serum of women with endometriosis. In the peritoneal fluid, the activity of CD4+ helper T cells are also decreased, which is in consistent with the fact that serum IL-10 levels are increased. There is also an increase in the proinflammatory and anti-inflammatory cytokines IL-1, IL-4, IL-6, IL-8, IL-10 and TNFα in the serum and in peritoneal fluid of women with endometriosis, particularly in advanced stages. Elevated levels of some growth factors, such as TGF-β, IGF-1, HGF, and VEGF were also described in patients’ peripheral blood. Due to local inflammation, the number of adhesions increases significantly in women with endometriosis, which can significantly contribute to the development of symptoms [25]. Some studies from innate immunity focused on changed macrophage forms and functions in the peritoneal cavity or in the blood stream. Activation of the CD36 receptor and matrix metalloproteases, which are required for the ability of phagocytosis, are strongly suppressed in endometriosis patients by significantly elevated levels of prostaglandin E2. Although the number of macrophages is increased and their cytokine products surge in volume and activation, these macrophages are differentiated more towards the M2 line and thus less suited to eliminate the endometrial tissue. Macrophages, deriving from monocytes, and neutrophil granulocytes, play a significant role in the development of inflammation and the immune response in the peritoneal environment; therefore, examining these cells from peripheral blood seems to be important [26,27]. It was also demonstrated that macrophages produce a significantly increased amount of proinflammatory cytokines, and there are data about defects in the phagocyte function [28,29]. Macrophages, natural killer cells and dendritic cells are present in peritoneal fluid with altered immunological function, which may be responsible for the residues of invaded menstrual debris [30]. However, it is still a question whether endometriosis develops due to such decreased elimination capacity and altered immunity, or the altered function of these cells are later developed as consequences of endometriosis.

Neutrophil granulocytes and monocytes are the less frequently examined representatives of the innate immune system; however, they play a vital role in the defense against tumors and infections, but also have a role in healthy and pathologic pregnancy [31,32,33]. Next to the numerical and functional changes of these cells in endometriosis, they may control the function of other immune cells as well. In experimental circumstances, it is shown that blood monocytes stimulate the endometrial cell proliferation, while the peritoneal macrophages inhibit it. At the same time, macrophage’s cytotoxic function is inversely correlated with the stage of the endometriosis, so the disturbed function is maintained in these cells, which is regulated by the prostaglandin synthesis [34,35,36]. It has been demonstrated that some monocyte-specific chemokines act differently if the cells from healthy patients are incubated in peritoneal fluid from patients with endometriosis [26].

Our hypothesis was that the neutrophil granulocytes and monocytes, as professional phagocytes, should play a role in the elimination of endometriosis lesions. In our previous work, we presented that in endometriosis patients the phagocyte function of both cell types is significantly decreased compared to the healthy controls. When examining the same individuals, after the complete surgical removal of the disease, phagocyte function has been normalized of both cells. At the same time, we ruled out the effect of surgical procedure on phagocyte function with the examination of a control surgical group (other benign indications than endometriosis), as there was no difference between the phagocyte function of the cells compared to the healthy womens’ results [18].

As a continuation of our previous work, a plasma experiment study was designed. In these experiments, we sought the answer to whether the decreased phagocyte function of the examined cells is reversible or not. At the same time, we examined the effect of autologous and heterologous (same examined group, but another individual’s sample) plasma on the phagocyte function and tried to find the answer for the heat sensitivity of the factor(s).

As phagocyte function is normalized in neutrophil granulocytes and monocytes not only after the surgical removal of endometriosis lesion, but also after incubating the cells from patients with endometriosis in the plasma samples of healthy women, it can be assumed that immunosuppressive factors are present in the peripheral blood of the patients, but the cells’ phagocyte function can be normalized in the absence of affected plasma; thus, the decreased phagocyte function is not irreversible. It can be concluded that the decreased phagocyte function is caused by such factor(s), which are in the peripheral blood. This hypothesis is supported with the observation, as the incubation of granulocytes and monocytes isolated from healthy women in the plasma of endometriosis patients, led to a significant decrease in the phagocyte function, whereas if the same cells were incubated in plasma from women after the complete removal of endometriosis, the phagocyte function did not change and remained on a normal level. Due to these results, we assume that the production of this immunosuppressant factor is produced by the endometriotic lesion itself, and without the lesion, the production of the factor ceases. That observation may also substantiate that endometriosis is a systemic disease which affects the peripheral immune cells as well, which may lead to systemic immunological consequences, such as increased risk of infection [30], in which depleted phagocyte function may play a role. Results from plasma samples exposed to heat give rise to the assumption that these immunosuppressive factors are heat resistant, since no significant differences were found between incubations in heat-inactivated and non-inactivated plasma in either group. As to the question whether the heterologous plasma medium affects the phagocyte function, no significant differences were found in either group, as we assume that the “foreign” environment does affect the phagocyte function.

The identification of this immunosuppressive factor in the peripheral blood of women with endometriosis can be a potential target for further research. We cannot rule out the possibility that, in addition to elevated proinflammatory cytokines, higher levels of anti-inflammatory cytokines (IL-10) in the blood of endometriosis patients may also affect phagocyte functions [25]. Based on our findings, we hypothesize that the elimination of endometrial cells in the abdominal cavity is impaired due to compromised immune functions, and the phenomenon outlined may be part of the pathogenesis of endometriosis.

The limitations of this study are the relatively low number of patients and the long-lasting experimental method. Our research may also shed light on the assumption that not only the dysfunction of the immune system is able to facilitate the development of endometriosis, but endometriosis itself can cause immune dysfunction. With these results, the possibility of medical therapy may come closer, either through the awakening of immunosurveillance or through the antagonist countering of systemic immunosuppressive factors.

These results highlight even more the generally accepted requirement of the complete surgical removal of endometriosis, as with proper surgical treatment, some of the immune dysfunction can also be restored.

## 5. Conclusions

Phagocyte function of neutrophil granulocytes and monocytes in endometriosis patients decreased significantly, but after complete surgical removal of the endometriosis, this function will normalize. This reduction in phagocyte function may cause by a heat-stable, peripheral immunosuppressant factor. By eliminating this factor from the environment of the cells, the phagocyte function will normalize again. Initially, normal phagocyte function of neutrophil granulocytes and monocytes can be disturbed if the cells are incubated in a plasma sample from an endometriosis patient. Further examinations are needed for the identification of this immunosuppressant plasma factor.

## Figures and Tables

**Table 1:** Clinical characteristics of patients with endometriosis and healthy women.

	Endometriosis Patients (*n* = 8)	Healthy Women (*n* = 16)	*p*-Value
**Age (year)**	33.38 (±4.57)	32.06 (±4.25)	NS
**BMI ^a^ (kg/m^2^)**	21.64 (±2.64)	20.78 (±2.27)	NS
**Gravidity ^b^**	1 (0–2)	1 (0–3)	NS
**Parity ^b^**	0 (0–1)	1 (0–3)	NS

Mean values ± standard deviations are presented. NS: not significant. ^a^ BMI: body mass index ^b^ Values are expressed as median (range).

## Data Availability

The data presented in this study are available by contacting the corresponding author.
